# Trans-thoracic versus trans-hiatal resection for oesophageal carcinoma: a retrospective comparative study of a single-centre case series

**DOI:** 10.1186/s43057-020-00035-y

**Published:** 2020-11-25

**Authors:** Aram Baram, Hiwa Sherzad

**Affiliations:** 1 Department of Surgery, School of Medicine, Faculty of Medical Sciences, University of Sulaimani, François Mitterrand Street, Sulaymaniyah, 46001 Iraq; 2 Department of Thoracic and Cardiovascular Surgery, Sulaimani Teaching Hospital, Al Sulaymaniyah, Kurdistan Region 46001 Iraq; 3 Kurdistan Board for Medical Specialization/Cardiothoracic and Vascular Surgery, Sulaimani DOH, Al Sulaymaniyah, Kurdistan Region Iraq

**Keywords:** Oesophageal cancer, Surgical resection, Operative approaches, Cancer-free survival, Long-term outcome

## Abstract

**Background:**

Oesophageal carcinoma (EC) is the eighth most common cancer. Surgery is the cornerstone of management for resectable EC. Trans-thoracic oesophagectomy (TTE) and trans-hiatal oesophagectomy (THE) are the two most widely practised procedures. Most of the related controversies are centred on both early and late post-operative complications and mortality (in terms of overall survival and cancer-free survival).

This was a single-centre, retrospective, comparative study analysing the outcomes of two EC resection methods. All 87 patients underwent surgery by the same surgical team over 13 years. Consequently, 87 oesophagectomies with curative intent were performed and divided into the TTE group (group A = 47) and the THE group (group B = 40).

**Results:**

The mean patient age was 65.60 ± 6.30 years in the TTE group and 63.48 ± 9.34 years in the THE group. No significant difference was found in operative time, blood loss or duration of stay in the intensive care unit. The duration of hospital stay was significantly different between the THE and TTE groups (17.25 ± 5.92 vs. 12.93 ± 3.44, respectively; *P* ≤ 0.001). In-hospital mortality was higher in the TTE group (9/47, 19.14%) than in the THE group (5/40, 12.5%) (*P* = 0.400). The mean survival rate from our series showed the superiority of group A (TTE) (65.56 months) over group B (THE) (45.01 months), with *P* = 0.146.

**Conclusion:**

No high level of evidence suggests the superiority of one surgical procedure over another. The THE procedure is less time-consuming concerning care and follow-up, and most patients were more satisfied and experienced less pain than with the TTE procedure. Both THE and TTE have comparable post-operative anastomotic complications, and they have no significant long-term survival differences.

## Background

Oesophageal carcinoma (EC) is the eighth most common cancer in the world and is the sixth leading cause of cancer-related mortality worldwide because of its high malignant potential and poor prognosis [1]. The incidence of EC is expected to increase by approximately 140% over the next 10 years, faster than any other solid cancer in the world [2]. EC affects more than 500,000 people worldwide annually [3]. The National Cancer Institute in the USA estimates approximately 18,440 new cases and approximately 16,170 deaths from EC in 2020 [4]. EC is four times more common in men than in women [1]. The incidence rate varies internationally by approximately 21-fold [5]; within the highest risk area, the rate is greater than 100 per 100,000 (Asian EC Belt) [2, 6].

Two main histopathological types of EC exist; together, they account for approximately 90% of all cases of EC: squamous cell carcinoma and adenocarcinoma [7].

Squamous cell carcinoma, the most common type of EC worldwide [1, 2, 6] and the most common subtype of EC in developing countries [8], evenly affecting the mid and lower oesophagus, has a strong correlation with smoking, alcohol consumption, nutritional deficiencies, chronic inflammation and irritation [2, 9]. Adenocarcinoma is the most common type of EC in Western countries; it has a strong predilection to the lower third of the oesophagus in up to three quarters of cases and is commonly associated with columnar-lined metaplastic epithelium (Barrett’s oesophagus), gastroesophageal reflux disease, smoking and obesity [6–9].

Although *Helicobacter pylori* (*H. pylori*) is a well-known risk factor for gastric carcinoma, no significant correlation between *H. pylori* infection and EC has been found in the general population, and it was even found to have a protective effect in oesophageal adenocarcinoma. However, such a correlation was found to be significant in the Middle East [10].

Surgery is the cornerstone of management for early-stage EC [11, 12]. The most common surgical techniques used to date are trans-thoracic oesophagectomy (TTE) (Ivor Lewis procedure) [13], trans-hiatal oesophagectomy (THE) (Orringer procedure) [14], three-field oesophagectomy (McKeown procedure) [15] and minimally invasive oesophagectomy [16].

While surgical resection is the only treatment modality with curative intent, many researchers have proved that nonsurgical management offers better survival benefits, that oesophagectomy has a relatively high index of mortality and that oesophageal cancer is an incurable disease [2].

Surgery still has a powerful impact on overall survival according to multivariable analyses. The death risk of patients treated with curative surgery is significantly lower than that of patients treated with definite chemoradiotherapy. Furthermore, for patients with stage T3N(+) and T4 disease, surgery combined with neoadjuvant treatment is associated with a significantly higher survival rate than surgery alone or definite chemoradiotherapy [11].

Patients who undergo surgery experience significantly longer survival than those who do not; therefore, curative resection should be considered for oesophageal cancer patients who are medically fit for surgery. Neoadjuvant treatment is recommended for surgically resectable stage T3-T4 EC [11].

A propensity-matched analysis showed that 525 patients who were given preoperative therapy followed by surgery had a median survival duration of 32.3 months compared with 21.9 months in patients who refused surgery. In a multivariate analysis, refusal of surgery remained a strong predictor of poor survival (odds ratio, 1.72; *P* < 0.001) [2].

Trans-thoracic oesophagectomy employs excision of the oesophagus through right thoracotomy under direct vision, excision of the tumour and peritumoural lymphatic tissues combined with mobilization of the stomach through midline laparotomy and reconstruction of the anastomosis in the posterior mediastinum [13]. As the patient undergoes both laparotomy and thoracotomy, there might be more post-operative cardiorespiratory complications, such as mediastinitis and sepsis, and post-operative anastomotic leaks, but this procedure provides more satisfactory oncological clearance of peritumoural tissue and lymph nodes [17–20].

Trans-hiatal oesophagectomy employs dilatation of the oesophageal hiatus through midline laparotomy, dissection of the oesophagus by blunt dissection in the posterior mediastinum without thoracotomy, and then, through a longitudinal left cervical incision, the oesophagus is exposed and gastroesophageal anastomosis is performed in the neck [14]. Therefore, it provides less possibility for compromised post-operative cardiorespiratory function and eliminates the risk of mediastinitis, but the oncological outcome is less favourable because of less oncological clearance [17–20].

Three-stage oesophagectomy (McKeown) combines laparotomy and right thoracotomy with cervical dissection and anastomosis [15]. The potential advantage of this procedure over the other two approaches is more comprehensive lymph node dissection and less need to extend the thoracotomy incision since the anastomosis is in the neck, and it avoids the morbidity associated with intrathoracic anastomosis [21, 22].

In minimally invasive oesophagectomy (MIE), laparoscopy and thoracoscopy are used for intraoperative staging, followed by gastric mobilization and intrathoracic oesophagectomy, respectively. Since it was first introduced by Cuschieri et al. in 1992 [16] as a subtotal endoscopic oesophagectomy through the right thoracoscopic approach, many institutions have reported using this technique in association with either laparotomy or thoracotomy or, more recently, with endoscopy, avoiding open techniques completely [23]. Although the initial results were promising, with a comparable outcome to open resection, avoiding thoracotomy will further reduce pulmonary complications associated with an open approach, and better visual control allows favourable oncological quality of resection. However, there is still no convincing evidence that MIE is superior to open oesophagectomy [24].

Considerable controversies exist about the optimal surgical approach. Most are centred on both early and late post-operative complications and mortality (in terms of overall survival and cancer-free survival).

Our study aimed to evaluate the differences in both early and late surgical outcomes of the most commonly performed procedures for oesophageal resection in our facility (trans-thoracic versus trans-hiatal oesophagectomy).

## Methods

### Study design

This was a single-centre, retrospective, comparative, observational study analysing the outcomes of the two most common methods of EC resection (trans-thoracic versus trans-hiatal oesophagectomy). Patients with retrospective data were included and analysed prospectively. This manuscript has been reported in line with the Strengthening the Reporting of Cohort Studies in Surgery (STROCSS) statement [25].

### Setting

This study was conducted in a single academic institution. Between February 2007 and February 2020, 87 oesophageal resections with curative intent were included: TTE (group A = 47) and THE (group B = 40).

All the data were obtained directly from the patients, patients’ relatives and medical records. Approval was granted from the Ethical and Scientific Council of our institution. This study was registered at ResearchRegistry.com (researchregistry5755).

Patient characteristics, including demographic data (Table 1), comorbidities, preoperative imaging data (barium swallow, abdominal ultrasound (US) and computed tomography (CT) of the chest and abdomen), cardiorespiratory function (pulmonary function test (PFT), echocardiography and chest radiography) and endoscopic variables from both oesophagogastroduodenoscopy (EGD) and endoscopic ultrasound (EUS), were analysed. As in the beginning of the study positron emission tomography-computed tomography (PET-CT) was not available, we depended on chest CT and EUS findings for the purpose of preoperative clinical staging.

**Table 1:** Patient characteristics

Variables	Category	Group A (TTE)	Group B (THE)	***P*** value
		***n*** (%) 47, (54.02%)	***n*** (%) 40, (45.97%)	
**Age (Mean ± SD)**	Year	65.60 ± 6.30	63.48 ± 9/34	0.212
**Age group**	40–50	1 (2.1%)	5 (12.5%)	
	51–60	9 (19.1%)	7 (17.5%)	
	61–70	29 (61.7%)	24 (60%)	
	71–80	8 (17%)	4 (10%)	
**Gender**	Male	30 (63.8%)	27 (67.5%)	0.720
	Female	17 (36.2%)	13 (32.5%)	
**Smoking**		26 (55.3%)	24 (60%)	0.660
**Comorbidities**	DM	19 (40.4%)	11 (27.5%)	0.206
	HTN	41 (87.2%)	31 (77.5%)	0.231
	IHD	25 (53.2%)	27 (67.5%)	0.175
	COAD	8 (17%)	14 (35%)	0.055+
**Clinical stages**	I	3 (6.4%)	1 (2.5%)	0.489
	II	20 (42.5%)	13 (32.5%)	
	III	24 (51.0%)	26 (65.0%)	
**Histology**	SCC	35 (74.4%)	24 (60%)	0.529
	Adenocarcinoma	12 (25.5%)	16 (40%)	

All patients underwent a histopathological examination for EC of the mid and lower oesophagus. Patients with distant metastases were excluded. Patients were staged according to the 8th edition of the TNM (tumour, node and metastasis) staging system.

Operative details, technique, safety margins, type of anastomosis, duration, intraoperative complications and blood loss were analysed.

Post-operative follow-up, intensive care unit stay, total hospital stay and any post-operative complications (wound infection, anastomotic leak and vocal cord paralysis) were recorded. Patients were followed up at regular intervals with an operative consultant every month for up to 3 months, then every 3 months for up to 1 year and then every 6 months for up to 5 years.

### Surgical procedures

Patients were thoroughly discussed at multidisciplinary meetings, and decisions regarding the surgical approach were made by the operating surgeon according to his experience and comfortability with the operative approach and patient characteristics (tumour stage, level and comorbidities).

In group A (47 patients), standard trans-thoracic oesophagectomy (Ivor Lewis operation) was performed. In group B (40 patients), standard trans-hiatal oesophagectomy (Orringer procedure) was performed. Standard gastric tubes were used in both groups. All gastroesophageal anastomoses were hand-sewn with a single layer of interrupted absorbable Vicryl (Ethicon Inc., USA). A stapler was not used for the anastomosis of suture lines in this series. No gastric drainage procedure was performed. Finally, a standard feeding jejunostomy tube was inserted into group A patients only. An intercostal drain was inserted into all patients in group A and inserted into select patients in group B. Cervical and abdominal wounds were always drained and closed with standard methods.

### Post-operative care

All efforts were made to wean the patient from mechanical ventilation in the immediate post-operative period. All the patients remained in the intensive care unit for a minimum of 24 h and were transferred to the conventional surgical ward accordingly. In cases of failed extubation, a further extubation attempt was made within the subsequent days. Enteral feeding started through the jejunostomy tube on the second or third post-operative day in group A and through the nasoentric tube in group B. Post-operative respiratory physiotherapy was encouraged in the form of incentive spirometry and early mobilization.

Patients were kept nil by mouth for at least 7 days. On the 8th post-operative day, if there were no clinical or radiological signs of a leak, then a liquid diet was allowed within the following days, while a cervical drain was kept in place.

### Statistical analysis

The data were collected and entered into an Excel sheet; after coding, they were transferred to Statistical Package for the Social Sciences (SPSS), version 22. IBM Corporation and R environment version 3.2.2 were used for data analysis. Descriptive and quantitative analyses were performed. The relationships between the initial findings and subsequent morbidity and mortality were determined using the chi-square test or Fisher’s exact test for nominal variables and Student’s *t* test for quantitative variables. A *P* value ≤ 0.05 was considered significant. Kaplan-Meier survival curves were used to estimate tumour-free survival.

## Results

A total of 87 patients underwent oesophagectomy for oesophageal cancer. Patients were grouped into group A (47 patients (54.02%) TTE) and group B (40 patients (45.97%) THE). Demographic details of both groups are shown in Table 1. Overall, there were no significant differences in demographic characteristics between the groups. The mean patient age was 65.60 ± 6.30 years in the TTE group and 63.48 ± 9.34 years in the THE group.

Male patients predominated in both groups. Chronic obstructive airway disease (COAD) was the most common associated comorbidity in group B (35%) (vs. 17% in group A; *P* = 0.055+). The most common clinical presentations were dysphagia (solid (96.6%) and liquid (89.7%)) and anorexia (62.1%). The majority of our patients were in stages II (37.9%) and III (57.4%). Three patients from group A had a lymph node (LN) status of N2 and received neoadjuvant chemotherapy (Tables 1, 2 and 3).

**Table 2:** Clinical stage distribution in two groups of patients studied

Tumour stage	Group A	Group B	Total	***P*** value
T1N0M0	3 (6.4%)	1 (2.5%)	4 (4.6%)	0.621
T2N0M0	3 (6.4%)	2 (5.0%)	5 (5.7%)	1.000
T2N1M0	9 (19.1%)	12 (30.0%)	21 (24.1%)	0.238
T2N2M0	1 (2.1%)	0 (0%)	1 (1.1%)	1.000
T3N0M0	12 (25.5%)	8 (20.0%)	20 (22.9%)	0.541
T3N1M0	17 (36.2%)	17 (42.5%)	34 (39.1%)	0.546
T3N2M0	2 (4.3%)	0 (0%)	2 (2.3%)	0.497
Total	47 (100%)	40 (100%)	87 (100%)	-

*P* = 0.557, not significant, Chi-square test

**Table 3:** Clinical stage distribution in two groups of patients studied

Clinical stage	Group A	Group B	Total	***P*** value
Stage I	3 (6.4%)	1 (2.5%)	4 (4.6%)	0.621
Stage II	10 (21.3%)	8 (20.0%)	18 (20.7%)	0.884
Stage IIB	10 (21.3%)	5 (12.5%)	15 (17.3%)	0.280
Stage III	23 (48.9%)	26 (65.0%)	49 (56.3%)	0.132
Stage IIIB	1 (2.1%)	0	1 (1.1%)	1.000
Total	47 (100%)	40 (100%)	87 (100%)	-

*P* = 0.489, not significant, Fisher exact test

The operative details and post-operative courses are shown in Table 4. Differences in operative time, total blood loss during the operation and number of hours of stay in the intensive care unit post-operatively were not significant between the groups. There was a statistically significant difference in the duration of hospital stay post-operatively among patients who received THE vs. TTE (17.25 ± 5.92 vs 12.93 ± 3.44, respectively) (*P* ≤ 0.001).

**Table 4:** Operative details and post-operative course

	Group A (TTE)	Group B (THE)	***P*** value
	***n*** (%), 47 (54.02%)	***n*** (%), 40 (45.97%)	
Duration of surgery (min)	172.77 ± 35,67	178.48 ± 36.14	0.462
Blood loss (ml)	619.15 ± 175.25	587.5 ± 234.45	0.474
Intraoperative complications	20 (42.6%)	10 (25%)	0.086
ICU stay (h)	28.51 ± 11.93	30.13 ± 15.86	0.590
Hospital stay (days)	12.93 ± 3.44	17.25 ± 5.92	< 0.001

Detailed post-operative complications are shown in Table 5. Hypokalaemia was the most common post-operative complication among TTE patients (57.4%) (versus 22.5% among THE patients; *P* = 0.001). Although statistically non-significant, we found more anastomotic leaks in the THE group (10/40 (27.5%)) than in the TTE group (11/47 (21.2%)) (*P* = 0.499). Re-operation was necessary in three patients who received TTE, and the stent was covered in the remainder of patients (7 patients), while 7 patients who received THE were treated conservatively with a T-tube and cervical wound care, and re-operation was performed in four patients. Vocal cord paralysis was noted in 3 patients in the THE group (7.5%) but not in any patient in the TTE group (*P* = 0.093).

**Table 5:** Post-operative complications

Complications	TTE	THE	***P*** value
	***n*** (%), 47 (54.02%)	***n*** (%), 40 (45.97%)	
Hypokalaemia	27 (57.4%)	9 (22.5%)	0.001
Wound infection	20 (42.5%)	18 (45%)	0.819
Anastomotic leak	10 (21.2%)	11 (27.5%)	0.499
Pulmonary complications	1 (2.12%)	2 (5%)	0.592
Cardiac complications	4 (8.5%)	2 (5%)	0.683
Vocal cord paralysis	0 (0%)	3 (7.5%)	0.093
In-hospital mortality	9 (19.14%)	5 (12.5%)	0.400
Post-operative dysphagia	7 (14.89%)	8 (20%)	0.530
Tumour recurrence	3 (6.38%)	6 (15%)	0.291

In-hospital mortality was higher in the TTE group (9/47, 19.14%) than in the THE group (5/40, 12.5%) (*P* = 0.400). Leak was the cause of death (mediastinitis-related sepsis) in 5/9 patients in the TTE group and in 3/5 patients in the THE group. Respiratory failure and inability to wean from a ventilator caused death in one patient in the TTE group (2.12%), while in the THE group, pulmonary complications (respiratory failure) were encountered in two patients (5%); one was a consequence of post-operative leaks (*P* = 0.592). Cardiac complications were noted in 4 (8.5%) patients in the TTE group, three of whom had acute myocardial infarction with sudden cardiac arrest and one who developed acute rapid atrial fibrillation (AF) and was treated medically with anti-arrhythmic medications. In the THE group, cardiac complications were noted in 2 patients (5%) (one with sudden cardiac arrest and another with rapid AF who was treated medically; *P* = 0.683).

Post-operative dysphagia was noted in both the TTE (7 (14.89%)) and THE (8 (20%)) groups (*P* = 0.530). The tumour recurrence rate was not different between the groups (TTE 3/47 vs. THE 6/40, *P* = 0.291).

**Fig. 1:**
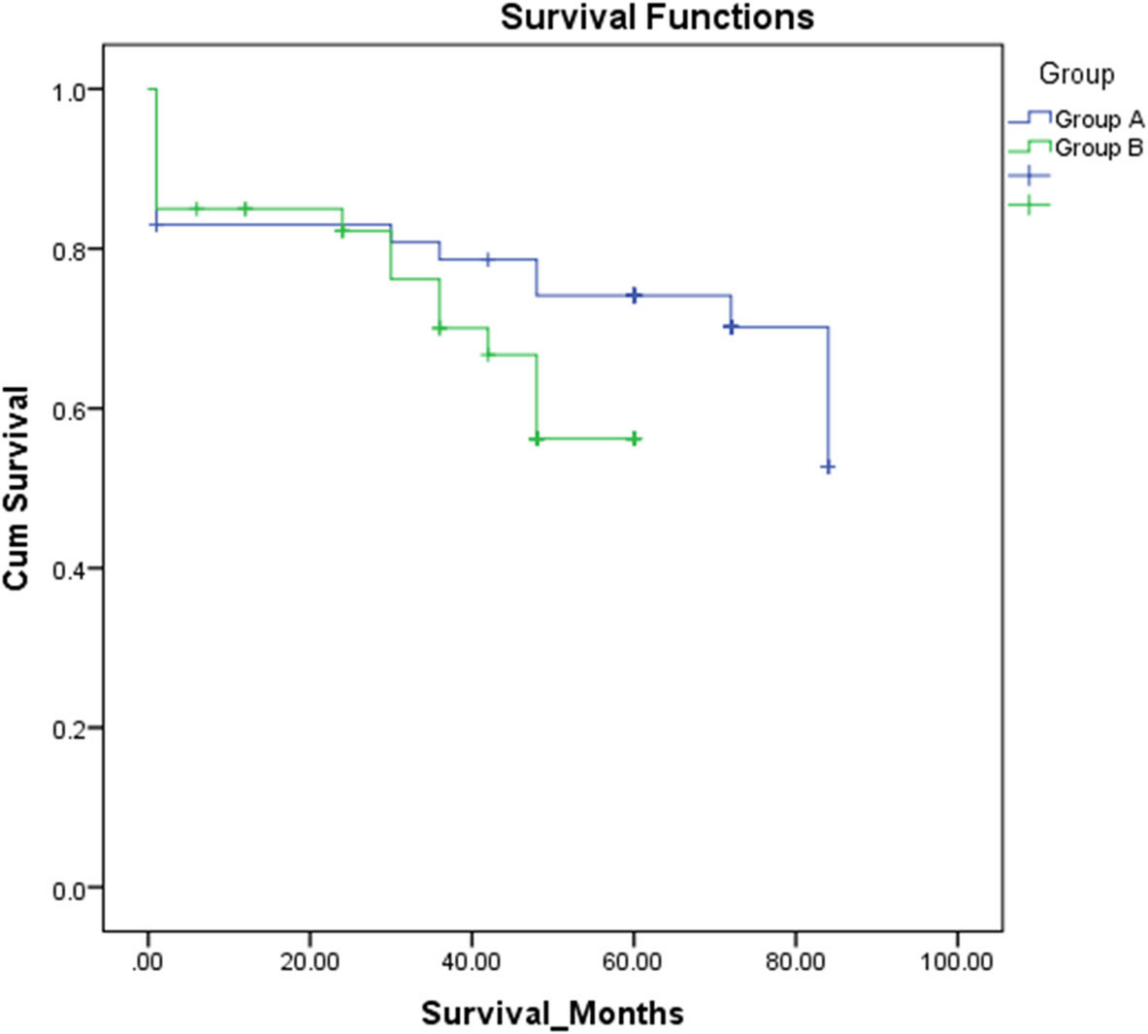
Kaplan-Meier survival curve shows 5-year survival in both the TTE (group A) and THE (group B) groups

The estimated survival duration from our series showed that the mean survival duration was longer in group A (TTE) (65.56 months) than in group B (THE) (45.01 months), with *P* = 0.146 (Fig. 1).

## Discussion

The exact incidence of EC in our locality (Iraqi Kurdistan) is unknown due to the lack of a national cancer registry; therefore, there is a strong need to perform research on different types of cancer in our region. The most credible published data from our single-province cancer registry (Hiwa Hospital) over 8 years of observation (2006–2014) showed that the age-specific incidence of EC is approximately 18.77 for males and 13.18 for females/100,000 population/year for those aged older than 65 years [26]. This high rate of incidence is expected, as Iraqi Kurdistan is included within the Asian Esophageal Cancer Belt [26].

EC is a highly aggressive tumour; half of the patients present later with distant metastasis, and only 30–40% of patients are operable at the time of presentation [1, 12]. Surgery is the cornerstone of management for resectable EC [11, 12]. There is no high level of evidence to suggest the superiority of one surgical procedure over another; most of the observations have been from individual studies and small-sample randomized controlled trials. Each surgical approach has its own pros and cons: some advocate TTE for oncological perspective only, while others advocate THE, as it is associated with relatively less early morbidity and mortality. The decision regarding surgical technique is based mostly on the experience of the surgeon and hospital, comorbidities, level and stage of the oesophageal tumour [11, 27]. The choice of approach was surgeon preference guided mainly by nodal staging, MDT, tumour size and extend. The surgical approach for each patient was thoroughly discussed at the multidisciplinary meetings, and decision regarding surgical approach was made by the operating surgeon according to his experience and comfort ability with the operative approach and patients characteristics (tumour stage, level, mediastinal nodal status and comorbidities)

In our series, there was no statistically significant difference between the groups at baseline in terms of demographic or tumour characteristics; however, the rate of COAD was higher in group B (35%) than in group A (17%; *P* = 0.055+). This higher number of COAD patients in the THE group can be explained by the fact that TTE is associated with higher post-operative cardiorespiratory compromise; therefore, selecting these high-risk patients for THE might eliminate or decrease post-operative respiratory complications [28].

Hulscher et al. [19] conducted a randomized controlled trial that reached level I of evidence regarding the best surgical treatment for EC. THE was associated with a shorter operative duration (3.5 vs. 6 h), less blood loss (1 vs. 1.9 litters), a lower perioperative morbidity rate (pulmonary complications 27% vs. 57%, chylous leakage 2% vs. 10%) and shorter hospital stay (mean 15 days vs. 19 days, *P* ≤ 0.001) than TTE. Within our series, we did not identify a statistically significant difference in operative time (172.77 ± 35.67 vs. 178.48 ± 36.14, respectively; *P* = 0.462), blood loss (619.15 ± 175.25 vs 587.5 ± 234.45, respectively; *P* = 0.474), perioperative morbidity such as pulmonary complications (2.12% vs. 5%, respectively; *P* = 0.592) or cardiac complications (8.5% vs. 5%, respectively; *P* = 0.683%), between the TTE and THE groups. Chylous leakage was not observed in either group. We observed higher respiratory complications in the THE group, although the difference was not statistically significant, but we found that COAD (in the THE group) was associated with other systemic diseases, especially cardiovascular diseases such as ischaemic heart disease and hypertension.

Early post-operative complications, such as hypokalaemia, were observed more often in the TTE group than in the THE group (57.4 vs. 22.5%, respectively; *P* = 0.001). These later post-operative complications were not reported by many authors; in our case, the possible explanation could be insufficient replacement or loss from the jejunostomy feeding tube we used for group A patients [29]. Concerning in-hospital mortality, although the difference was not statistically significant, the mortality rate was higher in the TTE group (19.14%) than in the THE group (12.5%) (*P* = 0400). Of those patients, 5/9 had anastomotic leak-related sepsis, three died from sudden cardiac arrest (acute myocardial infarction) and one died from respiratory failure. The higher complication rate in the TTE group has been well explained in the literature: a leak in the chest is associated with more severe clinical outcomes, such as mediastinitis and sepsis [28].

Patients who received TTE had a shorter hospital stay than patients who received THE (12.93 ± 3.44 vs. 17.25 ± 5.92, respectively; *P* ≤ 0.001). We correlated the longer hospital stay of the THE group in our series with anastomotic leaks, although the leak rate was comparable in our series. In contrast, post-operative leaks after TTE was associated with a more severe clinical course and longer hospital stay. However, in our series, leaks after TTE were managed with a covered stent (7/10), while early post-operative leaks were treated with re-operation. Leaks after THE were treated conservatively (7/11) with the controlled fistula technique, opening of the cervical wound and packing and daily dressing rather than with a stent because of the unavailability of a conventional stent at the cervical location [30].

Gooszen et al. [31] compared the surgical approach and predictors of post-operative anastomotic leaks. The anastomotic leak rate was 19.6%. They found a lower rate of anastomotic leaks with TTE (17.0%) than with THE (21.9) (*P* = 0.025) and that independent predictors of anastomotic leaks were ASA fitness grades III and IV, DM, COAD, history of cardiac arrhythmia and proximal location of the tumour. In our series, the leak rate was 24.1%. Although statistically non-significant, patients who received TTE had a lower rate of leaks than those who received THE (21.3% vs. 27.5%, respectively; *P* = 0.499). We could not find such a relation between these variables and the leak rate in our series.

The higher rate of leaks with THE can been explained, as the reconstruction of a long gastric conduit and narrow thoracic inlet can compromise the vascularity of the conduit, with subsequent ischaemia and venous congestion, necrosis and disruption of the anastomosis [32, 33]. Despite a lower leak rate and shorter hospital stay with TTE, some surgeons (including us) believe that THE with cervical anastomosis will allow a wider resection margin of the oesophagus and less severe post-operative complications [31, 34].

The left recurrent laryngeal nerve (LRLN) paresis rate in our series was 7.5% (TTE 0% vs. THE 7.5%, *P* = 0.095). This higher rate of RLN paresis after THE indicates that RLN is mainly at risk during cervical dissection and reconstruction of the anastomosis. The rate found in our study is comparable to that reported by Gooszen et al. (7.0%) [32] but lower than that reported by Rindani et al. (11.2%) [33], Hulscher et al. (13%) [19] and Liu et al. (10.3%) [35]. All observed that THE was associated with a higher incidence of LRLN paresis.

Post-operative anastomotic stricture with subsequent dysphagia was observed in 14.89% of patients in the TTE group and 20% of patients in the THE group (*P* = 0.530). Three of seven patients (42.8%) in the TTE group and 5/8 (62.5%) in the THE group required one or more sessions of endoscopic dilatation. None of our patients required surgical intervention. This higher rate of anastomotic stricture associated with THE has also been reported by other studies. Boshier et al. [11] conducted a meta-analysis and found a stricture rate of 25.1% among patients in the THE group versus 21.8% among patients in the TTE group. Rindani et al. [35] found a stricture rate of 28% among patients in the THE group vs. 16% among patients in the TTE group and Liu et al. found a stricture rate of 19.8% among patients in the THE group vs. 13.5% among patients in the TTE group [35]. Gastric conduit ischaemia with subsequent post-operative anastomotic leaks, stapled anastomosis, cardiovascular diseases and COAD were identified as risk factors [24, 31, 33, 36, 37].

The patterns of tumour recurrence were as follows: 3 patients (6.38%) in the TTE group experienced recurrence (2 local and 1 distant) and 6 patients (15%) in the THE group experienced recurrence (5 local and 1 distant) (*P* = 0.291). Although statistically non-significant in our series, this higher rate of recurrence has been reported in the literature and is the point of controversy among authors. Some believe that TTE allows better access to the posterior mediastinum, with extended en bloc dissection of all peritumoural tissue and lymphadenectomy, hence reducing the rate of loco-regional recurrence [19], while others consider mediastinal lymph node involvement as systemic micro-metastatic disease and, thus, extended resection will not change the outcome [38, 39].

Colvin et al. conducted a meta-analysis from 1950 to 2010 (five randomized controlled trials and one meta-analysis) and concluded that TTE may offer a superior 5-year survival rate in patients with limited positive lymph nodes. However, other authors reported no significant difference (Boshier et al.: TTE 26.6% versus THE 25.8, *P* = 0.84 [11]; Kawoosa et al.: TTE 32.76% versus THE 30.24%, *P* = 0.596 [39]; and Rindani et al.: TTE 26% versus THE 24% [33]). The Kaplan-Meier survival curve (Fig. 1) shows 5-year survival in both the TTE and THE groups. The mean survival duration was higher in group A (TTE) (65.56 months) than in group B (THE) (45.01 months), with *P* = 0.146 (log-rank test).

Overall, there is no high level of evidence to date to demonstrate the superiority of one surgical approach over another, and there are several concerns and advantages with each surgical approach (TTE and THE) that remain controversial. The decision to perform oesophagectomy with either technique depends on the preference and experience of the operating surgeon and baseline physiological characteristics such as comorbidities and level and stage of the tumour [40, 41].

Our study was conducted on a relatively small sample and was non-randomized. The duration of follow-up was relatively acceptable for most patients. A larger study with randomization is required for a stronger level of evidence.

## Conclusions

Oesophageal carcinoma is a rapidly increasing cancer worldwide, with a distinct geographical distribution. Surgery remains the cornerstone for curative treatment in the early stages of oesophageal carcinoma, although long-term survival is poor. Considerable debate exists over the best surgical approach based on perioperative morbidity and mortality and long-term survival. Trans-hiatal oesophagectomy is associated with a longer hospital stay, a higher rate of respiratory complications, post-operative anastomotic leaks, recurrent laryngeal nerve injury, post-operative strictures, subsequent dysphagia and recurrence. Long-term survival is higher with TTE.

## Data Availability

All our raw data available upon request

## Abbreviations

EC: Oesophageal carcinoma
TTE: Trans-thoracic oesophagectomy
THE: Trans-hiatal oesophagectomy
MIE: Minimally invasive oesophagectomy
STROCSS: Strengthening the Reporting of Cohort Studies in Surgery
US: Ultrasound
CT: Computed tomography
PFT: Pulmonary function test
EGD: Oesophagogastroduodenoscopy
EUS: Endoscopic ultrasound
TNM: Tumour, node and metastasis
SPSS: Statistical Package for the Social Sciences
COAD: Chronic obstructive airway disease
LN: Lymph node
AF: Atrial fibrillation
LRLN: Left recurrent laryngeal nerve paresis
